# A bag of stones found incidentally

**DOI:** 10.11604/pamj.2026.53.83.51341

**Published:** 2026-02-13

**Authors:** Chuah Yoen Young, Lee Wen-Tzong

**Affiliations:** 1 Department of Biological Sciences, National Sun Yat-Sen University, Kaohsiung, Taiwan,; 2 Division of Gastroenterology and Hepatology, Department of Internal Medicine, Ping Tung Christian Hospital, Pingtung, Taiwan,; 3 Department of Biomechatronics Engineering, National Pingtung University of Science and Technology, Pingtung, Taiwan,; 4 School of Dentistry, College of Dental Medicine, Kaohsiung Medical University, Kaohsiung, Taiwan

**Keywords:** Gallstones, abdominal pain, incidental

## Image in medicine

A 91-year-old woman presented with right flank pain for two days without fever, dysuria, trauma, or recent falls. Physical examination revealed no costovertebral angle tenderness. Laboratory tests showed an elevated C-reactive protein level of 3.6 mg/dL without leukocytosis. Urinalysis demonstrated pyuria and bacteriuria. She was treated empirically for acute pyelonephritis with intravenous cefoxitin, and urine culture later grew *Escherichia coli* sensitive to cephalosporins. Abdominal computed tomography (CT) revealed rupture of a right renal cyst. Incidentally, the gallbladder was filled with multiple small, round, hyperdense calculi, producing a striking “bag of stones” appearance. These findings were consistent with asymptomatic cholelithiasis. The patient's flank pain improved after a 7-day course of antibiotics. She was discharged in stable condition, and outpatient follow-up was unremarkable. Asymptomatic gallstones are commonly detected incidentally, and most patients remain symptom-free; therefore, expectant management is generally recommended. Prophylactic cholecystectomy is reserved for selected high-risk patients, such as those with porcelain gallbladder, large gallstones (>3 cm), anomalous pancreaticobiliary ductal junctions, gallbladder adenomas, or underlying hemolytic disorders.

**Figure 1:**
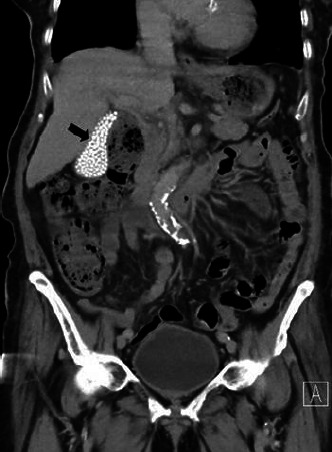
abdominal CT showing numerous small, round hyperdense gallstones filling the gallbladder, giving a characteristic “bag of stones” appearance (arrow)

